# Analysis of queuosine and 2-thio tRNA modifications by high throughput sequencing

**DOI:** 10.1093/nar/gkac517

**Published:** 2022-06-17

**Authors:** Christopher D Katanski, Christopher P Watkins, Wen Zhang, Matthew Reyer, Samuel Miller, Tao Pan

**Affiliations:** Department of Biochemistry and Molecular Biology, University of Chicago, Chicago, IL 60637, USA; Department of Biochemistry and Molecular Biology, University of Chicago, Chicago, IL 60637, USA; Department of Biochemistry and Molecular Biology, University of Chicago, Chicago, IL 60637, USA; Program of Biophysics, University of Chicago, Chicago, IL 60637, USA; Department of Medicine, University of Chicago, Chicago, IL 60637, USA; Department of Biochemistry and Molecular Biology, University of Chicago, Chicago, IL 60637, USA

## Abstract

Queuosine (Q) is a conserved tRNA modification at the wobble anticodon position of tRNAs that read the codons of amino acids Tyr, His, Asn, and Asp. Q-modification in tRNA plays important roles in the regulation of translation efficiency and fidelity. Queuosine tRNA modification is synthesized *de novo* in bacteria, whereas in mammals the substrate for Q-modification in tRNA is queuine, the catabolic product of the Q-base of gut bacteria. This gut microbiome dependent tRNA modification may play pivotal roles in translational regulation in different cellular contexts, but extensive studies of Q-modification biology are hindered by the lack of high throughput sequencing methods for its detection and quantitation. Here, we describe a periodate-treatment method that enables single base resolution profiling of Q-modification in tRNAs by Nextgen sequencing from biological RNA samples. Periodate oxidizes the Q-base, which results in specific deletion signatures in the RNA-seq data. Unexpectedly, we found that periodate-treatment also enables the detection of several 2-thio-modifications including τm^5^s^2^U, mcm^5^s^2^U, cmnm^5^s^2^U, and s^2^C by sequencing in human and *E. coli* tRNA. We term this method **p**eriodate-dependent **a**nalysis of **q**ueuosine and **s**ulfur modification sequencing (PAQS-seq). We assess Q- and 2-thio-modifications at the tRNA isodecoder level, and 2-thio modification changes in stress response. PAQS-seq should be widely applicable in the biological studies of Q- and 2-thio-modifications in mammalian and microbial tRNAs.

## INTRODUCTION

Among the ∼50 modifications in human RNA, queuosine (Q) is unique in that it requires a gut microbial and diet dependent metabolite for its installation. Q is a 7-deaza-7-aminomethyl-cyclopentenediol derivative that replaces the unmodified guanine at the wobble anticodon position (34 in tRNA nomenclature) of Tyr, His, Asn, and Asp tRNAs (1). Bacteria synthesize Q-modified tRNAs in an 8-step *de novo* biosynthesis pathway (2). In mammals, Q-modified tRNAs are derived from scavenging the queuine nucleobase, a catabolic product of the Q-modified tRNA in gut microbiome or diet. In humans, G34 is replaced with queuine, in these 4 tRNAs, by the heterodimeric enzyme guanine-tRNA transglycosylase (TGT) (3).

Although Q-modification in tRNA is not essential for life (for example, germ-free mice can have a normal life in the laboratory with a full synthetic diet lacking queuine (4)), Q34 is known to enhance decoding speed, tune decoding accuracy in translation, and modulate tRNA fragment biogenesis (5–9). In mammals, gut availability of queuine affects virulence of resident gut microbes (10) and modulates cancer growth (11,12). Q-modification levels in tRNA are especially high in human brain tissues (13) and queuine also plays a role in resistance to cancer metabolism (11) and neuronal damage (14). Since queuine must be scavenged from the gut and Q-tRNA modification is directly involved in protein biosynthesis, queuine and Q-tRNA modification present a clear connection between the gut microbiome and host proteostasis.

Currently, the methods of detecting and quantifying Q-modification in tRNA include radioactive guanine exchange, liquid chromatography-mass spectrometry (LC/MS), acryloylaminophenyl boronic acid (APB) or acid denaturing gel electrophoresis (15–17). Guanine exchange is only useful to quantify the total Q levels in all tRNAs. LC/MS can precisely analyze Q-modification in tRNAs, but it is difficult to apply it to tRNA isodecoders at a large scale. Gel electrophoresis methods can quantify Q-modification in individual tRNAs, but still requires micrograms of total RNA and is done for one tRNA species at a time. Neither LC/MS nor gels have sufficient resolution for the studies of tRNA isodecoders in mammals, which are tRNA sequences that share the same anticodon but differ in the body of the tRNA.

Thio-modifications are also widespread in mammalian and bacterial tRNAs (18). They include direct substitutions of the oxygen atom with sulfur at either the 2- (2-thio, s^2^) of C/U or 4- (4-thio, s^4^) position of U, and at 2-position of A (2-methylthio-isopentenyl-A, ms^2^i^6^A). In human tRNAs, the s^2^ modification occurs in the wobble anticodon uridine (U34) of several tRNAs. In *E. coli* tRNAs, the s^2^ modification occurs in the wobble anticodon uridines of several tRNAs, as well as position 32 of cytosine (s^2^C32) in the anticodon loop of several other tRNAs; furthermore, many *E. coli* tRNAs also contain the s^4^U modification at position 8 between the acceptor and D stem. The anticodon s^2^U34 modifications are generally, but not always accompanied by additional modifications at C5, and the thio-modification plays a crucial role in decoding efficiency of C- versus U-ending codons of Gln, Glu, Lys and AGY codons of Arg (19). The s^2^C32 modification plays a role in selective decoding of Arg codons (20). The s^4^U8 modification is a UV sensor that initiates UV response in *E. coli* (21).

Nextgen sequencing has become a versatile tool to study many RNA modifications. The modifications themselves, or derivatives from various chemical or enzymatic treatments, can result in distinctive signatures of base misincorporations (mutations), deletion, insertions, or stop in the RNA-seq data (22–24)—all caused by how reverse transcriptase reads the unique chemical structure of modification. To our knowledge, Q-modification does not readily leave a detectable signature in standard RNA-seq procedures, despite the large chemical moiety attached to the 7-position of the Q-base.

**Figure 1.**
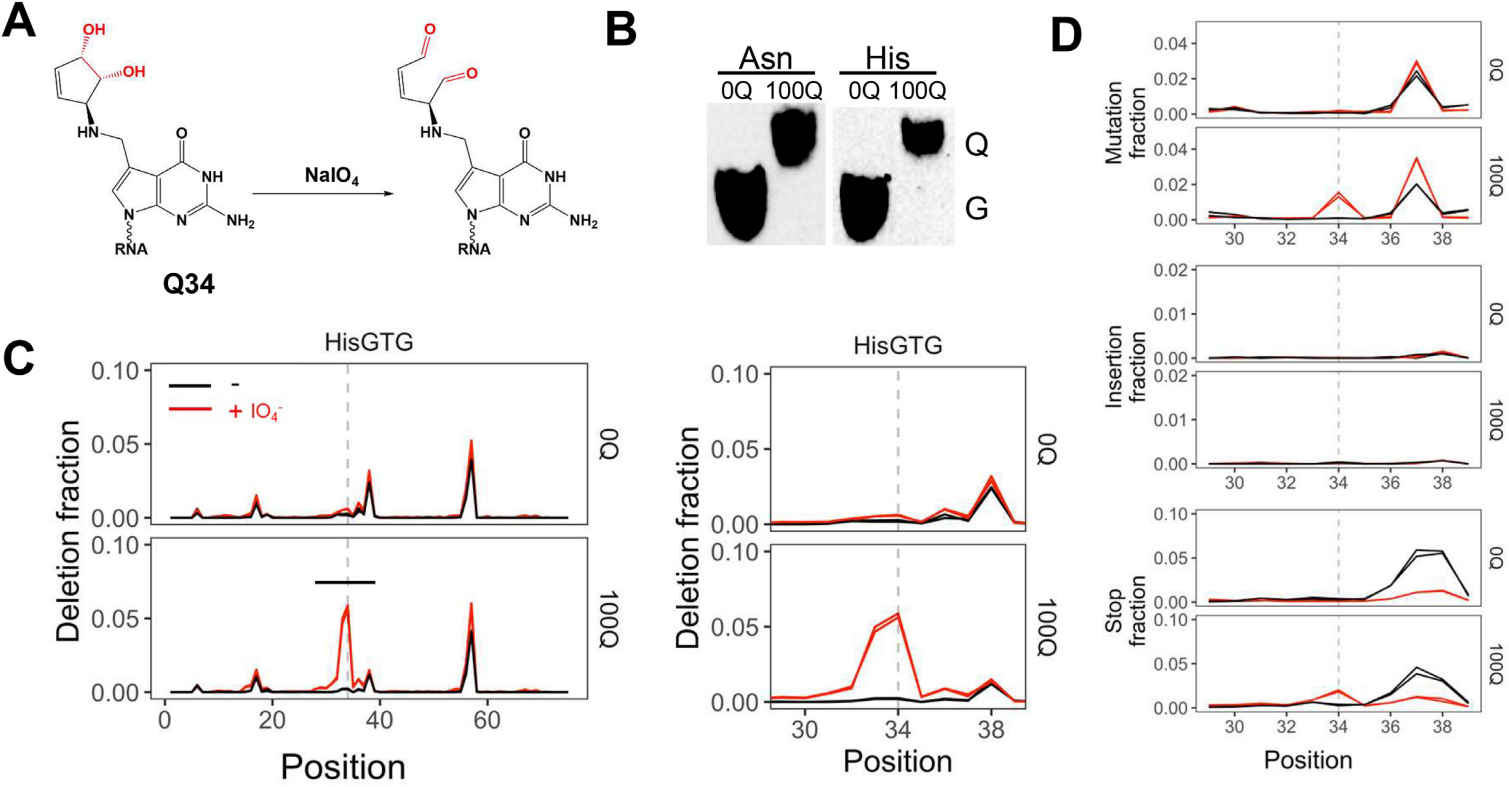
Q-modification in tRNA generates deletion signature after periodate treatment. (**A**) Chemical structure of the Q-base and its proposed periodate oxidized form. (**B**) Northern blot of APB gel showing the tRNA^Asn^ and tRNA^His^ Q-levels in 0Q and 100Q samples used in sequencing. (**C**) Deletion fraction of tRNA^His^, in 0Q and 100Q samples, ±periodate. Expanded view in the region of ±5 nt to Q34 residue (dashed line). The biological replicates are overlaid in each graph. Only the most abundant tRNA^His^ isodecoder is shown. A known modification that also produces deletion signature is m^1^G at position 37. (**D**) Same samples and legend as panel C showing mutation, insertion, and stop fractions in the region of ±5 nt to Q34 residue.

Here, we report that periodate treatment of the total RNA sample before reverse transcription produces new and quantitative sequencing signature of Q-modification in tRNA at single base resolution. It is well established that periodate can oxidize the *cis*-diol in the Q-base (Figure 1A). We found that upon mapping of the high throughput sequencing data to the respective reference tRNA, periodate-treated tRNAs show base skipping (deletion) at the Q-modified nucleotide. Q-modification levels can be semi-quantified using a calibration established with tRNAs without Q-modification (0Q) and full Q-modification (100Q). Unexpectedly, we also found that periodate treatment generates a strong sequencing signature for 2-thio tRNA modifications. Thus periodate treatment of modifications enables the investigations of both Q and 2-thio tRNA modifications.

## MATERIALS AND METHODS

### HEK293T cell growth

HEK293T cells were cultured with complete DMEM medium under normal conditions. 0Q HEK293T cells were obtained by culturing the cells with dialyzed FBS for certain passages, and 100Q HEK293T cells were obtained by treating 0Q cells with 1 μM queuine for 24 h (8). Briefly, HEK293T cells were grown in complete DMEM medium (Cytiva Hyclone SH30022.01) with 10% dialyzed FBS (Thermo Fisher Scientific 26400044) and 1% Penicillin-Streptomycin (Thermo Fisher Scientific 15070063) to 80% confluency and passaged. TRIzol reagent (Thermo Fisher Scientific 15596026) was used to extract total RNAs at each passage by following the manufacturer's manual. Q levels in tRNA^His/Asn^ were constantly examined at each passage by APB gel based Northern blot. Q modification fractions of tRNA^His/Asn^ dropped to below detection after ∼10 passages, these cells are designated as 0Q. 100Q cells were obtained by culturing 0Q cells to 60–80% confluency followed by incubation with 1 μM queuine for 24 h.

### Northern blot of APB gels

Northern blots were performed as previously described (12). Three μg of total RNA was added to each microcentrifuge tube and diluted to 9 μl with H_2_O. 1 μl of 1M Tris–HCl (pH 9.0) was added to the tube with mixing followed by incubation at 37°C for 30 min to deacylate tRNAs. 10 μl 2× RNA loading dye (8 M urea, 0.1 M EDTA, 0.05% Bromophenol blue, 0.05% Xylene cyanol) were added to each tube. All samples were loaded to a pre-run hand-cast 10% denaturing PAGE gel containing 0.5% (g/ml) Acrylamidophenylboronic acid (APB). The gel was run at 18W for ∼2–3 h until the xylene cyanol band was ∼1–2 cm to the bottom in the 4°C cold room using 1× TAE buffer. The gel area containing the target tRNAs was saved and the slightly larger sized Hybond-XL membrane (GE Healthcare, RPN303S) was put on top of the gel to take the gel out of the plate with caution. Dry RNA transfer was then performed using a gel dryer (Bio-Rad, 1651745) for 4 h at 80°C. The gel and membrane were separated by soaking in distilled water. The RNA was crosslinked to the membrane by UV for two times (254 nm, 1200 mJ). The membrane was then blocked for 30 minutes twice with hybridization buffer (20 mM phosphate, pH 7, 300 mM NaCl, 1% SDS) at room temperature. The membrane was incubated with 50 ml 3 pmol/ml biotinylated tRNA probes for 16 h at 60°C in the UVP Hybridizer Oven (Analytik Jena 95-0030-01) followed by washing with 50 ml washing buffer (20 mM phosphate, pH 7, 300 mM NaCl, 2 mM EDTA, and 0.1% SDS) for 30 min twice in the UVP Hybridizer Oven. The membrane was then incubated with streptavidin-HRP conjugate (Genscript M00091) in 30 ml hybridization buffer (1:5000–1:10 000 dilution) for 30 min at room temperature followed by three washes for 5 min each in 25 ml washing buffer. The membrane was then transferred to plastic wrap with the RNA-side facing up. Peroxidase detection reagent 1 and 2 (Bio-Rad 1705061) were mixed (0.1 ml per 1 cm^2^ membrane) and applied to the top of the membrane by pipetting. The membrane was incubated with the reagent mixture for 5 min under dark. The membrane was then transferred to a new plastic wrap to remove extra detection reagent. The membrane was scanned using the ChemiDoc imaging system (Bio-Rad) and the data was analyzed using ImageLab.

The oligonucleotide probe sequences were: tRNA^His^: 5′-biotin-TGCCGTGACTCGGATTCGAACCGAGGTTGCTGCGGCCACAACGCAGAGTACTAACCACTATACGATCACGGC; tRNA^Asn^: 5′-biotin-CGTCCCTGGGTGGGCTCGAACCACCAACCTTTCGGTTAACAGCCGAACGCGCTAACCGATTGCGCCACAGAGAC.

### *E. coli* growth and RNA extraction

*E. coli* MG1655 cells were grown in LB to a *A*_600_ of 0.4 before subjecting to the stress conditions. Cells were harvested by centrifuging 25 ml culture for 1 min at 12 000 rcf and decanting media. Mock treated cells, 25 ml, were left to grow for 10 min. Iron depletion stress was done by adding to 25 ml cells 2,2′-dipyridl (DIP) to 250 μM final concentration for 10 min. Hydrogen peroxide stress was done by adding H_2_O_2_ to 25 ml cells to a final concentration of 0.5% for 10 min. Glucose phosphate stress was done by adding to 25 ml cells α-methyl glucoside-6-phosphate (αMG) to a final concentration of 1 mM for 10 min. Cells were resuspended in 0.5 mL ice cold lysis buffer (150 mM KCl, 2 mM EDTA, 20 mM HEPES pH 7.5) then flash frozen in liquid nitrogen. RNA was extracted by a hot acid-phenol protocol. Briefly, 0.5 ml of acid-buffer phenol (pH 4.5 citrate) was added to frozen samples. Samples were incubated in a heat block with shaking at 50°C for 30 min. The aqueous phase was extracted for another round of phenol extraction and two rounds of chloroform extraction before ultimately precipitating with glycoblue, 300 mM sodium acetate, and 3 volumes of ethanol. Samples were incubated for 1 hour at -80°C, then centrifuged at maximum speed (20k RCF) for 45 min to pellet RNA. Pellets were washed twice with 70% ethanol, then resuspended in water.

### Periodate treatment

#### One-pot deacylation and β-elimination for tRNA charging

For RNA that was periodate treated, up to 500 ng of total RNA in 7 μl was used for optional one-pot beta-elimination prior to library construction. To start, 1 μl of 90 mM sodium acetate buffer, pH 4.8 was added to 7 μl input RNA. Next, 1 μl of freshly prepared 150 mM sodium periodate solution was added for a reaction condition of 16 mM NaIO_4_, 10 mM NaOAc pH 4.8. Periodate oxidation proceeded for 30 min at room temperature. Oxidation was quenched with addition of 1 μl of 0.6 M ribose to 60 mM final concentration and incubated for 5 min. Next 5 μl of freshly prepared 100 mM sodium tetraborate, pH 9.5 was added for a final concentration of 33 mM. This mixture was incubated for 30 min at 45°C. To stop β-elimination and perform 3′ end repair, 5 μl of T4 PNK mix (200 mM Tris–HCl pH 6.8, 40 mM MgCl_2_, 4 U/μl T4 PNK, from New England Biolabs) was added to the reaction, and incubated at 37°C for 20 min. T4 PNK was heat inactivated by incubating at 65°C for 10 min. This 20 μl reaction mixture can be used directly in the first bar-code ligation by adding 30 μl of a ligation master mix described below.

#### Standard tRNA deacylation

For RNA that was not periodate treated, total RNA was prepared for library construction by first deacylating in a solution of 100 mM Tris–HCl, pH 9.0 at 37°C for 30 min, then neutralized by addition of sodium acetate, pH 4.8 to a final concentration of 180 mM. Deacylated RNA was then ethanol precipitated and resuspended in water or desalted using a Zymo Research Oligo Clean-and-Concentrator spin column.

#### On-bead periodate treatment

During library preparation (described below), RNA samples that underwent standard tRNA deacylation were treated with periodate when immobilized on streptavidin beads. Beads were resuspended in final concentration of 50 mM NaIO_4_, 0.1 M NaOAc/HOAc, pH 5, then incubated at room temperature for 30 min. Reaction was quenched with 10 μl 1 M ribose for 5 min. Beads were washed twice with high salt wash buffer.

### Sequencing library preparation

Libraries were prepared as described in multiplex small RNA sequencing (MSR-seq) paper (25). Briefly, after deacylation, tRNAs were ligated to a biotinylated hairpin oligo with a barcode and Illumina sequencing primer binding sites. Samples were incubated overnight at 16°C in a 50 μl ligation solution with the following final concentration: 15% PEG 8000, 1× NEB T4 RNA ligase I buffer, 50 μM ATP, 5% DMSO, 1 mM hexaamine cobalt (III) chloride, 0.8 μM barcode ligation oligo, and 1 U/μl NEB T4 RNA ligase I. After overnight ligation, 50 μl of 100 mM EDTA were added to each sample to inactivate the ligase. The samples were then combined and 8 μl of ThermoFisher Streptavidin MyOne C1 Dynabeads per sample were added to the combined samples. The biotinylated samples were allowed to bind the beads for 15 min. After binding, the beads were magnetized, the supernatant was removed, and the samples were washed once with a high salt Tween wash buffer (1 M NaCl, 0.1% Tween 20, and 20 mM Tris–HCl, pH 7.4) and once with low salt wash buffer (100 mM NaCl, 20 mM Tris–HCl, pH 7.4). The RT reaction was done with Superscript IV at 55°C for 10 min, then further incubated at 37°C overnight. Beads were then resuspended into 50 μl of RNase H master mix containing 0.4 U/μl RNase H (NEB) and 1× NEB RNase H buffer and incubated at 37°C for 15 min, then washed. Unextended hairpin-oligo was blocked by addition of 10 μl of 250 mM freshly prepared sodium periodate, 0.5 M sodium acetate, pH 5 were added to the RNase H digested sample and incubated at room temperature for 30 min. Afterwards, ribose was added to a final concentration of 167 mM to quench excess periodate at room temperature for 5 min. Beads were resuspended into 50 μl of a ligation master mix with the following components: 2 U/μl T4 RNA ligase I (NEB), 1x NEB T4 RNA ligase I buffer, 2 μM second ligation oligo, 25% PEG 8000, 50 μM ATP, 7.5% DMSO and 1 mM hexaamine cobalt chloride. After incubation at room temperature overnight (>12 h), the reaction was diluted with 50 μl water to reduce viscosity, washed once with high salt wash buffer and once with low salt wash buffer, and then resuspended in water. These beads were used as template for PCR reaction to make libraries for sequencing.

### Calibration samples

The queuosine calibration samples were mixed using a combination of 0% queuosine (queuosine-depleted) HEK total RNA and 100% queuosine (queuosine-abundant) HEK total RNA to a final volume of 10 μl. (Queuosine modification levels were quantified by Northern blot.) The calibration samples ranged from 0% queuosine to 100% queuosine in 10% intervals.

*S*amples on beads from the barcode ligation and multiplexing above were resuspended in 40 μl of deionized, autoclaved water and 10 μl of a 0.25 M NaIO_4_, 0.5 M NaOAc/HOAc, pH 5 (final concentration: 50 mM NaIO_4_, 0.1 M NaOAc/HOAc, pH 5) were added. The reaction proceeded at room temperature for 30 minutes and was quenched by addition of 10 μl of 1 M ribose for 5 min. After quenching, the samples were washed as stated in the Barcode ligation and multiplexing section.

### Reverse transcriptase screen

Libraries were prepared as above with standard deacylation conditions for three replicates of 100Q samples, however each sample was used in two different ligation reactions with different barcodes. After ligation, samples were pooled into two groups, differing only by the barcode used for each replicate. One group was further treated with periodate on bead as described above, while the other was not treated. Next all samples were combined – use of 6 barcodes allows differentiation of the three samples with periodate treatment and the three without. This combined mixture was used for further library construction. Before reverse transcription, the sample was divided into six aliquots, each used for reverse transcription with a different RT enzyme. The enzymes used were AffinityScript (Agilent 600105), HIV-RT (Worthington Biochemical Corporation, LS05003), MuMLV (NEB M0253L), RevertAid (ThermoFisher EP0441), SuperScript III (ThermoFisher 18080093), and SuperScript IV (ThermoFisher 11756050). Each RT product was kept separate for the remainder of library construction and PCR amplified with a different index for sequencing.

### Data analysis

Libraries were sequenced on Illumina Hi-Seq or NEXT-seq platform. First, paired end reads were split by barcode sequence using Je demultiplex with options BPOS = BOTH BM = READ_1 LEN = 4:6 FORCE = true C = false ^6^. BM and LEN options were adjusted for samples with a 3 nt barcode instead of 4, and for samples where the barcode is located in read 2. Barcode sequences are available on Github at https://github.com/ckatanski/Q_paper. Next read 2 files were used to map with bowtie2 (26) with the following parameters: -q -p 10 –local —no-unal. Reads were mapped to curated list of non-redundant tRNA genes with tRNAScane score >40 for respective organisms (human and *E. coli*). Bowtie2 output sam files were converted to bam files, then sorted using samtools. Next IGV was used to collapse reads into 1 nt window. IGV output.wig files were reformatted using custom python scripts (available on GitHub at https://github.com/ckatanski/Q_paper). The bowtie2 output Sam files were also used as input for a custom python script using PySam, a python wrapper for SAMTools (27) to sum all reads that mapped to each gene. Data was visualized with custom R scripts (available on GitHub at https://github.com/ckatanski/Q_paper). ‘Reads per million’ normalization was calculated by dividing the number or reads mapped to a specific gene by the total number of tRNA-mapped reads in that sample, and scaling by a factor of 1 000 000. Unless otherwise stated, analysis was limited to genes and positions with read coverage >100 reads. For presentation, the position value of each tRNA gene was adjusted to match canonical tRNA numbering (anticodon in positions 34, 35, 36). For calibration curve, Origin was used to fit linear or semiology line of best fit using least squares regression and calculated *r*^2^ statistics. Comparing change in this modifications during stress, an unpaired two-sided Wilcox test (Mann–Whitney): ns indicates a *P*-values >0.05, * <0.05, ** <0.01, *** <0.001.

## RESULTS AND DISCUSSION

### Periodate treatment produces deletion signatures for Q-modification in sequencing

Periodate is known to oxidize *cis*-diol groups into aldehydes. Periodate oxidation can be used to study tRNA aminoacylation levels by chromatography, microarrays, or sequencing (e.g. (28–30)). The Q-base has a *cis*-diol group that is a known substrate for periodate oxidation, a common reaction used to confirm the presence of Q-modification in APB gel electrophoresis (15,17). In our tRNA-seq procedure to measure tRNA charging levels, periodate treatment is a pre-requisite step in the sequencing library construction before reverse transcription (30,31). Using total RNA from HEK293T cells (Figure 1B) that are either completely devoid of tRNA Q-modification (0Q, (8)) or fully modified with Q (100Q), we found unexpectedly that tRNA^His^ from 100Q cells produced deletion in the high throughput sequencing data at the Q34 nucleotide at ∼5% level only in the periodate treated, but not in the untreated sample (Figure 1C). The signature is absent in 0Q cells. The periodate-dependent deletion signature of the Q nucleotide in sequencing is the most pronounced among the other signatures analyzed such as misincorporation of bases (mutation), addition of extra bases (insertion), or the location of the 5′ end of the sequencing products (stop; Figure 1D).

To further evaluate how a fully Q-modified nucleotide only results in a ∼5% deletion signature, we explored how different reverse transcriptases (RT) interact with periodate-oxidized-queuosine. Among six different RT enzymes tested, the Q-associated, periodate-dependent signature from mutation, deletion, or stops are highly idiosyncratic (Supplementary Figure S1A). Each enzyme leaves a distinct misincorporation signature composed of different fractions of mutations, deletions, insertions, and stops (Supplementary Figure S1B). The deletion fraction generated by the RT used in this work, SuperScript IV (SSIV), produced the most robust signal among the RTs tested.

**Figure 2.**
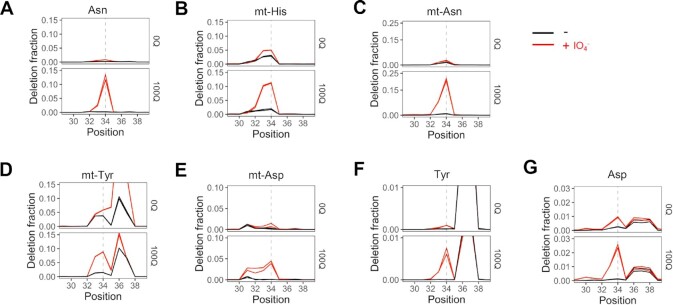
Periodate-treatment dependent deletion signatures in other tRNAs containing Q or glycoQ modifications. Shown are regions ±5 nt to the Q34 residue (dashed line) in each tRNA. Biological replicates are overlaid in each graph. For nucleus-encoded tRNAs, only the most abundant isodecoder for Asn/Tyr/Asp is shown. All residue numbers are according to the standard tRNA nomenclature, i.e. the wobble anticodon nucleotide is at position 34. (**A**) tRNA^Asn^, (**B**) Mitochondria-encoded tRNA^His^. (**C**) Mitochondria-encoded tRNA^Asn^. (**D**) Mitochondria-encoded tRNA^Tyr^. A known modification that also produces deletion signature is ms^2^i^6^A at position 37. (**E**) Mitochondria-encoded tRNA^Asp^. (**F**) tRNA^Tyr^. A known modification that also produces deletion signature is m^1^G at position 37. (**G**) tRNA^Asp^. The periodate-independent deletion signature at positive 37 is unknown.

A total of eight tRNAs in human cells can be modified with Q. The nucleus-encoded tRNA^His^ and tRNA^Asn^ are modified with Q, whereas tRNA^Tyr^ and tRNA^Asp^ are further modified to galactosyl-Q and mannosyl-Q, respectively (32,33). In addition, the mitochondria-encoded tRNAs for these same 4 amino acids are also modified with Q (34). We first examined the deletion signatures for other Q-modified tRNAs. The cytosolic tRNA^Asn^ displayed a relatively high deletion fraction of ∼13% at the Q34 position that is Q-modification and periodate-dependent (Figure 2A). All four mitochondrial tRNAs show deletion signatures in the same manner at the Q-modified nucleotide as well, ranging from ∼4% in mt-tRNA^Asp^ to ∼20% to mt-tRNA^Asn^ (Figure 2B–E). These results confirm that Q-modified nucleotide can indeed be detected using periodate-treated RNA-seq libraries.

Why does oxidized Q-base induce a deletion signature in the reverse transcriptase (RT) reaction in the sequencing library construction? Q-base affects anticodon-codon pairing through altering anticodon loop geometry and increasing its rigidity (35–37). *In vitro* studies of codon-anticodon complexes show a 3-fold increase in stabilization of Q–U pairings over G-U, while pairings with C were destabilized (35). The 5-member ring of the Q-base is located in the major groove of the RNA-DNA hydrid in the active site of reverse transcriptase. Periodate oxidation opens the ring which may lead to increased flexibility and steric occupancy of the oxidized moiety in the major groove, thereby inducing the RT to skip the oxidized Q nucleotide. However, the deletion fraction has a strong dependence on the context of the Q-modified nucleotide (Supplementary Figure S1C–E). One empirical factor is the nucleotide sequences immediately upstream of the Q34 residue. tRNA^Asn^ has an upstream C32 (5′GGCUQUU) and a high deletion fraction, whereas tRNA^His^ has an upstream U32 (5′CGUUQUG) and a low deletion fraction. Mitochondrial mt-tRNA^Asn^ (5′AGCUGUU) has C32 and mt-tRNA^His^ (5′GAUUGUG) has U32 which again is consistent with the observed high and low deletion fraction for these two tRNAs. Mitochondrial mt-tRNA^Asp^ (5′CUUUGUC) has U32 and U31 which may correlate with the observed deletion signature spanning four nucleotides (Figure 2E). Furthermore, other modifications near the Q34 nucleotide may also generate additional context dependence of our results. For example, mt-tRNA^Tyr^ (5′GACUGUA) could have a high deletion fraction, but this may be obscured by the ms^2^i^6^A37 modification that has a much larger deletion signature with and without periodate treatment (Figure 2D).

The glyco-Q modified tRNAs do not significantly react with the boronic acid derivative to cause a gel shift like the Q-modified tRNAs. However, both galactose and mannose can form a small proportion of furanose tautomer that contain *cis*-diol in equilibrium with their major pyranose tautomer (38). Our 100Q sample is known to have nearly stoichiometric amount of glycosylated Q-modification as measured by a combination of APB and acid denaturing gel electrophoresis (12). We found that both nucleus-encoded tRNA^Tyr^ and tRNA^Asp^ also display deletion signatures using periodate treatment in our sequencing (Figure 2F, G), albeit the deletion fraction was only ∼0.5% or 2% for tRNA^Tyr^ and tRNA^Asp^, which are substantially lower than Q-modified tRNAs. Since the deletion background in our sequencing is <0.1%, these low deletion fractions are still useful in detecting glyco-Q modifications, especially in tRNA sequencing where the coverage at the glyco-Q nucleotides can easily reach >1000. The differences in the deletion fraction among tRNA^Tyr^ and tRNA^Asp^ may be related to the periodate reacted product of galQ and manQ, both tRNAs have C32 in their upstream sequences (tRNA^Tyr^ 5′GACUGUA, tRNA^Asp^ 5′GCCUGUC).

### Deletion fraction can be used to quantify Q-modification levels

**Figure 3.**
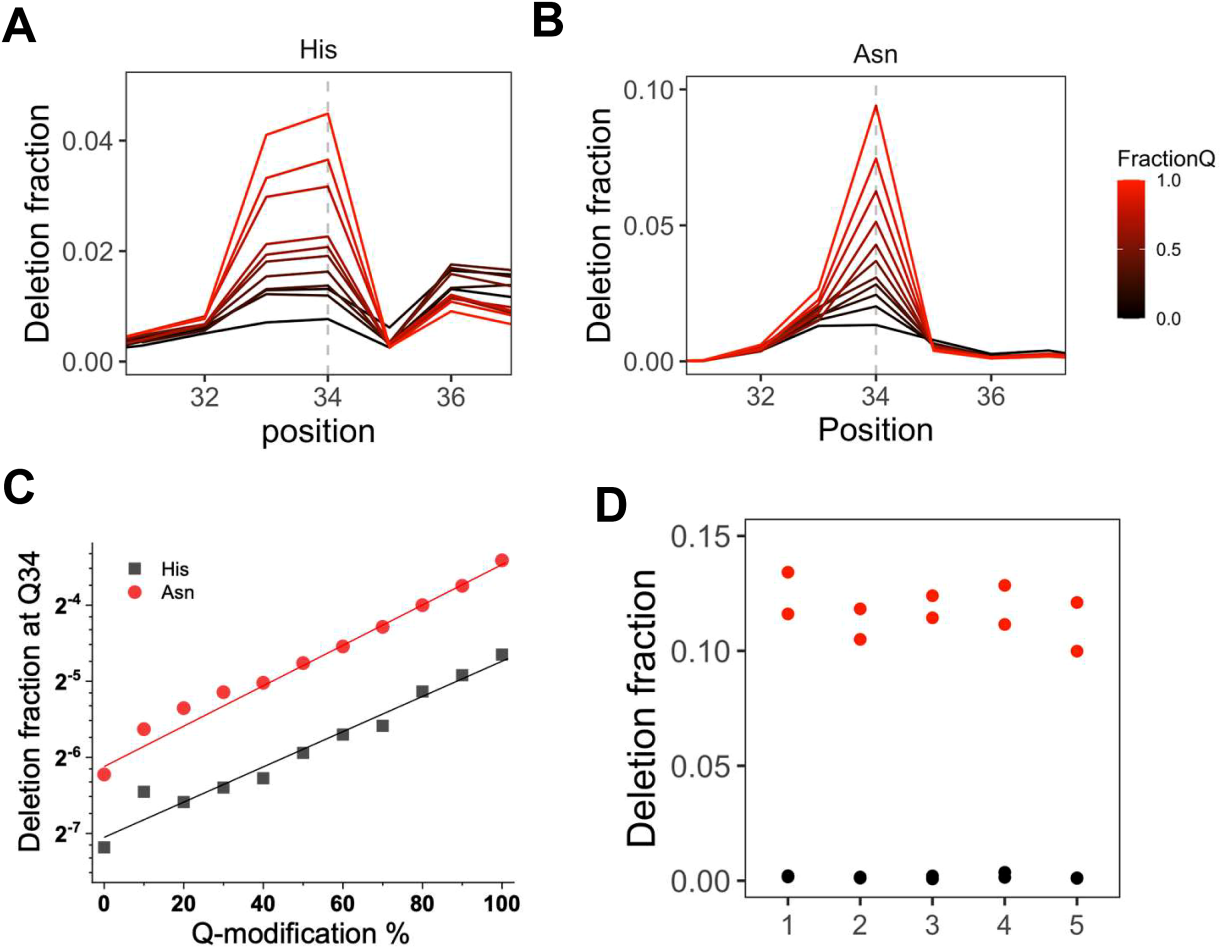
Quantitative assessment of Q-modification levels in tRNA^His^ and tRNA^Asn^. Shown are regions ±3 nt to the Q34 residue (dashed line) in each tRNA. (**A**) Overlay of deletion fraction for tRNA^His^ of the 11 samples that are pre-mixed with proportions of 0Q and 100Q RNAs. (**B**) Overlay of deletion fraction for tRNA^Asn^ of the 11 samples that are pre-mixed with proportions of 0Q and 100Q RNAs. (**C**) The deletion fraction at Q34 can be fit to the equation log_2_*y* = *a* + *bx*, where *a* is the intercept and *b* is the slope. The tRNA^His^ curve has a fit of *a* = –7.0, *b* = 0.023 and *r*^2^ = 0.964. The tRNA^Asn^ curve has a fit of *a* = –6.0, *b* = 0.025, and *r*^2^ = 0.985. (**D**) Isodecoder Q levels of the top 5 expressed tRNA^Asn^.

An important application to use sequencing to study Q-modification is the potential ability to quantify Q-modification fraction in any biological sample, which enables simultaneous assessment of transcriptome-wide tRNA properties associated with Q-modification. To assess whether the deletion signature can be used to quantify Q-levels, we systematically mixed two biological samples of 0Q and 100Q HEK293T cells at varying ratios between 0 and 100% and performed sequencing reactions after periodate treatment. As expected, the deletion fraction at the Q34 position for tRNA^His^ and tRNA^Asn^ steadily increases between the mixture with increasing proportion of 100Q RNA (Figure 3A, B). Changes in the deletion fraction has a better fit with an exponential dependence of Q-modification fraction (r^2^ = 0.964, 0.985, Figure 3C) than linear fits (*r*^2^ = 0.901, 0.923, Supplementary Figure S2). A non-linear fit can be explained by Q34 tRNA being reverse transcribed less efficiently than unmodified (G34) tRNA upon periodate treatment. Because only up to 20% of Q34 tRNA produces deletion, the quantitative production of the deletion-containing cDNA is skewed when the Q-level is low. Similar results have been observed for other modifications that reduce the RT efficiency such as N1-methyl-A (m^1^A) compared to unmodified RNA (39,40).

Interestingly, both tRNA^His^ and tRNA^Asn^ show a very similar slope in the Q-level calibration curve (Figure 3C). This result is consistent with the absolute deletion fraction being dependent on the sequence context of Q34 in individual tRNAs, but the changing Q-levels respond the same way to the RT reaction.

Five tRNA^Asn^ isodecoders comprise >95% of total tRNA^Asn^ (Figure 3D), whereas a single tRNA^His^ isodecoder comprises >99% of total tRNA^His^ in our HEK293T RNA samples. Examination of the deletion fraction of the five tRNA^Asn^ isodecoders in 100Q samples show nearly identical deletion rates, indicating all are modified at the same level, which is consistent with the identical sequence in the 11 nucleotide window around the Q34 residue (Supplementary Figure S1B).

### Periodate treatment also produces sequencing signatures in 2-thio-modifications

**Figure 4.**
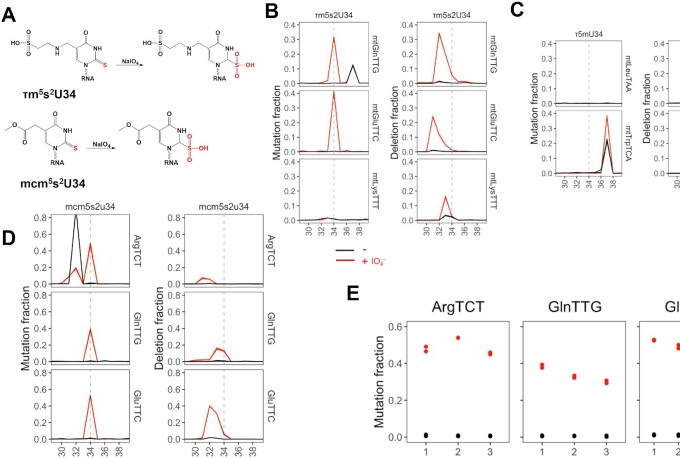
Periodate-treatment dependent analysis of 2-thio tRNA modifications. All residue numbers are according to the standard tRNA nomenclature, i.e. the wobble anticodon nucleotide is at position 34. (**A**) Chemical structure of the 2-thio-modifications and their proposed periodate oxidized forms. Shown in (B–D) are mutation and deletion signatures in regions ±5 nt to the relevant residue (dashed line) in each human tRNA, 0Q samples with (red) and without (black) periodate treatment. Biological replicates are overlaid in each graph. (**B**) Mitochondrial tRNA^Gln^, tRNA^Glu^, and tRNA^Lys^ known to contain 5-taurinomethy-2-thio-U (τm^5^s^2^U) at wobble anticodon position. (**C**) Mitochondrial tRNA^Leu^(TAA), and tRNA^Trp^ known to contain 5-taurinomethy-U (τm^5^U) at wobble anticodon position. (**D**) Nucleus-encoded tRNAs known to contain 5-methoxycarbonylmethyl-2-thio-U (mcm^5^s^2^U)34. A known modification that also produces signatures is m^3^C at position 32 for tRNA^Arg^(TCT). (**E**) mcm^5^s^2^U34 mutation rates and abundance for isodecoders of tRNA^Arg^(TTC), tRNA^Gln^(TTG) and tRNA^Glu^(TTC) with and without periodate treatment.

Periodate is also known to oxidize sulfides and thiol groups (41–43) which could alter the sequencing signatures of thio modifications in tRNA. Thio modifications in human tRNAs are primarily 2-thio (s^2^), which substitutes the oxygen atom with a sulfur at the 2-position of uridine (Figure 4A). The 2-thio modification is in the minor groove of the DNA–RNA hybrid in the active site of the reverse transcriptase. 2-thio oxidation may alter the proof-reading mechanism of the RT, resulting in detectable signatures in RNA sequencing.

In mitochondria-encoded tRNAs, the s^2^U-modification is present in the wobble anticodon position of tRNA^Gln^, tRNA^Glu^ and tRNA^Lys^ in the context of 5-taurinomethyl-2-thiouridine (τm^5^s^2^U, (34)). We found a strong mutation signature for mt-tRNA^Gln^ and mt-tRNA^Glu^ at the modified nucleotide that are also accompanied with a strong double deletion signature 1-2 nucleotides upstream from the modified nucleotide that are periodate-dependent but not Q-dependent, as expected (Figure 4B, Supplementary Figure S3A). Mitochondrial tRNA^Lys^ shows periodate-dependent deletion consistent with a 2-thio modification but no mutation (Figure 4B, Supplementary Figure S3A). This result may be derived from the unique sequence and/or other modifications around the 5-methyltaurine modified nucleotide. Among the τm^5^s^2^U34 modified tRNAs, only mt-tRNA^Lys^ has a N6-threonylcarbamoyladenosine (t^6^A) modification at position 37 (34), which may influence obtaining a mutation signature in the RT reaction. Another possibility is that the mt-tRNA^Lys^ in our specific sample (total RNA from HEK293T cells) may not contain a 2-thio modification at the U34 position.

Importantly, the two mitochondrial tRNAs that have 5-methyltaurine, but no 2-thio modification, mt-tRNA^Leu^(TAA) and mt-tRNA^Trp^ (34), do not show periodate-dependent mutation or deletion signature at the τm^5^U position (Figure 4C), which lends support that the periodate-dependent mutation and deletion in mt-tRNA^Gln^ and mt-tRNA^Glu^ are indeed derived from the 2-thio modification. Unexpectedly, strong, periodate-dependent mutation and deletion signatures are present in mt-tRNA^Trp^ that corresponds to the known ms^2^i^6^A37 in this tRNA. The sulfur atom in ms^2^i^6^A may also be subject to the thio-modification, which likely contributes to the periodate-dependent signatures in sequencing.

In nucleus-encoded tRNAs, the s^2^-modification is present in the wobble anticodon position of tRNA^Arg^(TCT), tRNA^Gln^(TTG) and tRNA^Glu^(TTC) in the context of 5-(carboxy)methylaminomethyl-2-thiouridine (mnm^5^s^2^U34). Indeed, we found a strong mutation signature right at the modified nucleotide for all three tRNAs in both 0Q and 100Q cells that is periodate-dependent, but not Q-dependent (Figure 4D, Supplementary Figure S3B). A double deletion signature is also present on or upstream of the modified nucleotide depending on the tRNA species. These results indicate that periodate treatment is capable of detecting mnm^5^s^2^U34 modifications, although the absolute mutation and deletion signatures likely depend on the context of the neighboring sequences and other modifications.

We compared the mutation signatures for the abundant tRNA^Arg^(TCT), tRNA^Gln^(TTG) and tRNA^Glu^(TTC) isodecoders in our samples (Figure 4E, Supplementary Figure S3C). Among the isodecoders of tRNA^Arg^(TCT) and tRNA^Gln^(TTG), the mutation rates are comparable to each other despite the variations of their abundances. This result may be expected as the sequence differences of these isodecoders are all outside of the ±5 nucleotide window of the modification (Supplementary Figure S4). A ∼2.5-fold difference was observed among the tRNA^Glu^(TTC) isodecoders. This difference, however, may be attributed to the substantial sequence or other modification difference between these isodecoders within the ±5 nucleotide window of the thio-modification (Supplementary Figure S4), rather than a biological difference of the mnm^5^s^2^U34 modification fraction.

### Thio-modifications in *E. coli* and in stress response

**Figure 5.**
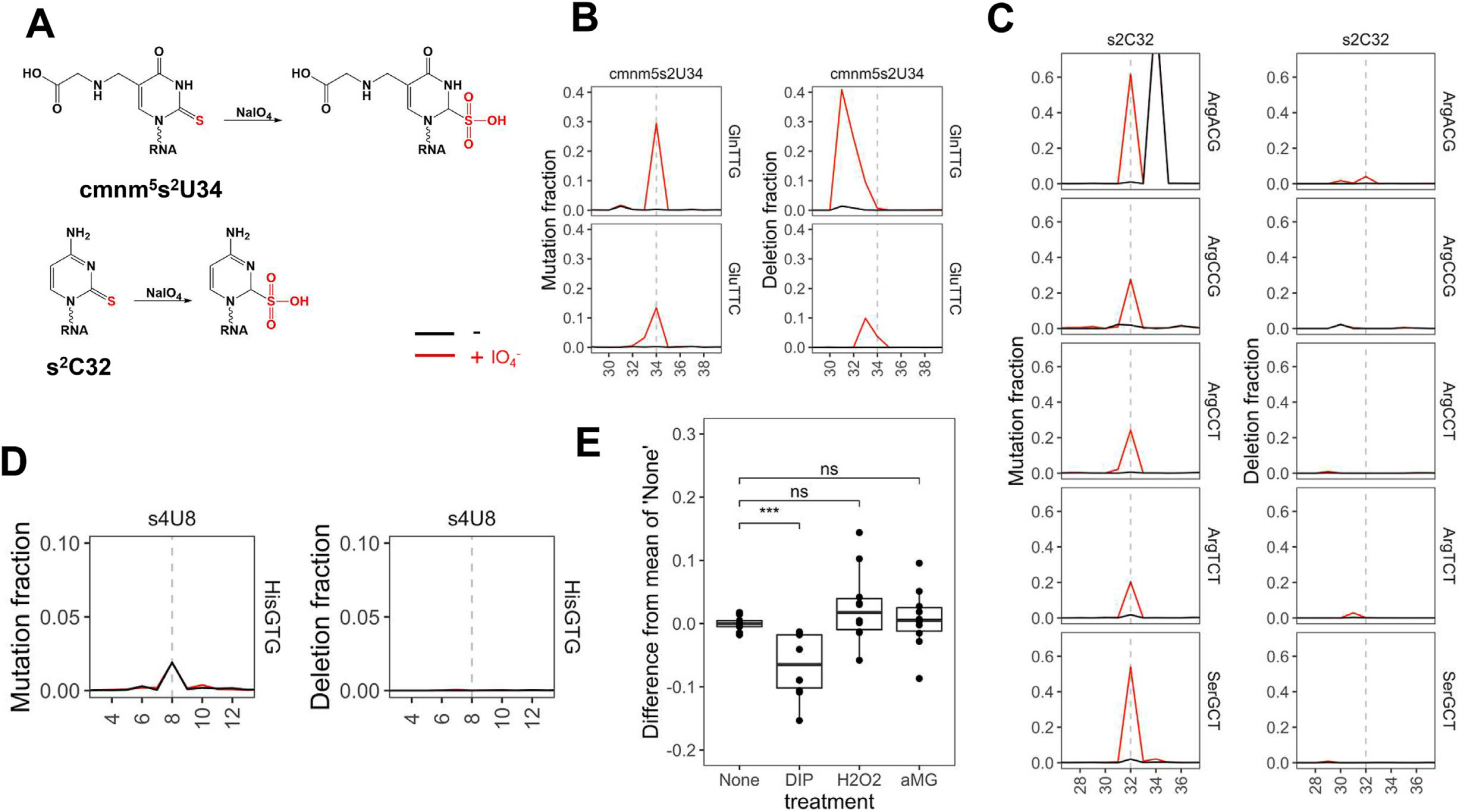
2-thio tRNA modifications in *E. coli* tRNA and response to stress. Shown are mutation and deletion signatures in regions ±5 nt to the relevant residue (dashed line) in each tRNA. (**A**) Chemical structure of the 2-thio-modifications and their proposed periodate oxidized forms. (**B**) tRNA^Gln^(TTG) and tRNA^Glu^(TTC) known to contain 5-carboxymethylaminomethyl-2-thio-U (cmnm^5^s^2^U) 34. (**C**) tRNA^Arg^(ACG), tRNA^Arg^(CCG), tRNA^Arg^(CCT), tRNA^Arg^(TCT), and tRNA^Ser^(GCT) known to contain 2-thio-C (s^2^C) at position 32. A known modification that also produces signatures is I34 in tRNA^Arg^(ACG). (**D**) tRNA^His^ known to contain 4-thio-U (s^4^U) at position 8. (**E**) s^2^C32 response to 2,2′-dipyridyl (DIP), hydrogen peroxide (H_2_O_2_), and methyl α-D-glucopyranoside (αMG), biological replicates are shown in each graph. ***P < 10^–3^, ns: not significant.

2-Thio modifications are also present in *E. coli* tRNAs (Figure 5A). We performed sequencing of *E. coli* tRNA with and without periodate treatment, and found strong, periodate-dependent mutation and deletion signatures for the known 5-carboxymethylaminomethyl-2-thiouridine (cmnm^5^s^2^U) modification at the wobble anticodon position in tRNA^Gln^(TTG) and tRNA^Glu^(TTC) (Figure 5B). Again, the mutation is at the modified nucleotide, whereas the deletion is at or immediately upstream of the modification nucleotide.

*E. coli* tRNA contains 2-thio-C (s^2^C) and 4-thio-U (s^4^U) modifications that are absent in human tRNA. We found strong, periodate-dependent mutation for the known s^2^C32 modification in all 5 tRNAs right at the modified nucleotide (Figure 5C). In each case, a low level of deletion was also observed at 1–3 nucleotides upstream of the s^2^C modified nucleotide. On the other hand, s^4^U modification at position 8 only shows an expected mutation signature (44) that is independent of periodate treatment and no deletion signature (Figure 5D). The 2-thio modification is located in the minor groove, whereas the 4-thio modification is in the major groove of the DNA–RNA hybrid in the active site of RT. These results are consistent with thio-oxidation in the minor groove interfering with the proof-reading activity of reverse transcription.

To examine the biological response of 2-thio-modifications, we subjected *E. coli* to the 2,2′-dipyridyl (DIP) stress, which chelates Fe^2+^, oxidative stress with H_2_O_2_, or glucose starvation with αMG and performed tRNA-seq of the control and stressed samples. The mutation rates for the cmnm^5^s^2^U34 modified tRNA^Gln^(TTG) and tRNA^Glu^(TTC) did not change under these stress conditions (Supplementary Figure S5). In contrast, the s^2^C32 levels in all tRNAs were reduced in the DIP stress but not in H_2_O_2_ or αMG stress (Figure 5E). 2-Thio-C modification is installed by the enzyme TtcA which contains an iron-sulfur cluster in the active site (45), the s^2^C32 reduction under iron chelation is consistent with a reduction of the TtcA activity. It remains to be determined whether the reduction of s^2^C32 level affects decoding of specific codons (CGN and AGN) read by these modified tRNAs.

## CONCLUDING REMARKS

In summary, we found that periodate treatment of total RNA samples before reverse transcription enables the detection and semi-quantitation of Q-modified tRNAs through periodate-dependent deletion signature in high throughput sequencing. Even though the deletion fractions are not high, the sequencing coverage of tRNA can easily reach hundreds to thousands per nucleotide position so that even glyco-Q34 and mitochondrial Q-modifications can be assessed. Although Q-modification can be precisely quantified using boronate affinity electrophoresis (APB gel) or LC/MS, these methods cannot simultaneously measure the effects of Q-modification on other tRNAs in the cell to study tRNA fragment biogenesis and linkage to the tRNA abundance and modification at the transcriptome level. These previously hidden, Q-modification dependent properties can now be assessed using the PAQS-seq approach.

We also found that periodate treatment of RNA samples enables detection of 2-thio-modifications in tRNA by high throughput sequencing. Like Q-modification, the ability to analyze 2-thio-modifications together with their association with other tRNA properties in the cell should reveal new biological insights on the tRNA transcriptome. At this time, we do not have a calibration curve of 2-thio-modifications for more precise quantitation, although these can be readily obtained upon chemical synthesis of oligonucleotides containing these modifications. Nevertheless, the ability to study these modifications by sequencing when comparing biological samples from different conditions can already examine the biological response involving these modifications. The 2-thio-modification generated mutation fraction in sequencing is sufficiently high to enable its application to identify new modification sites in low abundant mRNAs.

Chemical and enzymatic treatment of RNA samples before RT reaction has proven to be an extremely versatile tool for RNA modification studies (46,47). We now add periodate treatment to this list for the studies of Q and 2-thio-modifications. As periodate treatment is part of the procedure to quantify tRNA charging by sequencing (30,31), Q and 2-thio-modification detection and semi-quantitation are already present in these sequencing data. Both deletion and mutation have low background in the standard sequencing results so that these modifications can be studied at high sensitivity.

## DATA AVAILABILITY

The sequencing data has been deposited to NCBI GEO under the accession number GSE196016.

## Supplementary Material

gkac517_Supplemental_File

## References

[1] Fergus C. , Barnes D., Alqasem M.A., Kelly V.P. The queuine micronutrient: charting a course from microbe to man. Nutrients. 2015; 7:2897–2929. 25884661 10.3390/nu7042897 PMC4425180

[2] Hutinet G. , Swarjo M.A., de Crecy-Lagard V. Deazaguanine derivatives, examples of crosstalk between RNA and DNA modification pathways. RNA Biol. 2017; 14:1175–1184. 27937735 10.1080/15476286.2016.1265200 PMC5699537

[3] Chen Y.C. , Kelly V.P., Stachura S.V., Garcia G.A. Characterization of the human tRNA-guanine transglycosylase: confirmation of the heterodimeric subunit structure. RNA. 2010; 16:958–968. 20354154 10.1261/rna.1997610 PMC2856889

[4] Marks T. , Farkas W.R. Effects of a diet deficient in tyrosine and queuine on germfree mice. Biochem. Biophys. Res. Commun. 1997; 230:233–237. 9016755 10.1006/bbrc.1996.5768

[5] Dixit S. , Kessler A.C., Henderson J., Pan X., Zhao R., D’Almeida G.S., Kulkarni S., Rubio M.A.T., Hegedusova E., Ross R.L. et al. Dynamic queuosine changes in tRNA couple nutrient levels to codon choice in Trypanosoma brucei. Nucleic Acids Res. 2021; 49:12986–12999. 34883512 10.1093/nar/gkab1204 PMC8682783

[6] Meier F. , Suter B., Grosjean H., Keith G., Kubli E. Queuosine modification of the wobble base in tRNAHis influences ‘in vivo’ decoding properties. EMBO J. 1985; 4:823–827. 2988936 10.1002/j.1460-2075.1985.tb03704.x PMC554263

[7] Tuorto F. , Legrand C., Cirzi C., Federico G., Liebers R., Muller M., Ehrenhofer-Murray A.E., Dittmar G., Grone H.J., Lyko F. Queuosine-modified tRNAs confer nutritional control of protein translation. EMBO J. 2018; 37:e99777. 30093495 10.15252/embj.201899777 PMC6138434

[8] Wang X. , Matuszek Z., Huang Y., Parisien M., Dai Q., Clark W., Schwartz M.H., Pan T. Queuosine modification protects cognate tRNAs against ribonuclease cleavage. RNA. 2018; 24:1305–1313. 29970597 10.1261/rna.067033.118 PMC6140461

[9] Muller M. , Legrand C., Tuorto F., Kelly V.P., Atlasi Y., Lyko F., Ehrenhofer-Murray A.E. Queuine links translational control in eukaryotes to a micronutrient from bacteria. Nucleic Acids Res. 2019; 47:3711–3727. 30715423 10.1093/nar/gkz063 PMC6468285

[10] Nagaraja S. , Cai M.W., Sun J., Varet H., Sarid L., Trebicz-Geffen M., Shaulov Y., Mazumdar M., Legendre R., Coppee J.Y. et al. Queuine is a nutritional regulator of *Entamoeba histolytica* response to oxidative stress and a virulence attenuator. mBio. 2021; 12:e03549-03520. 33688012 10.1128/mBio.03549-20 PMC8092309

[11] Hayes P. , Fergus C., Ghanim M., Cirzi C., Burtnyak L., McGrenaghan C.J., Tuorto F., Nolan D.P., Kelly V.P. Queuine micronutrient deficiency promotes Warburg metabolism and reversal of the mitochondrial ATP synthase in HeLa cells. Nutrients. 2020; 12:871. 10.3390/nu12030871 PMC7146442 32213952

[12] Zhang W. , Xu R., Matuszek Z., Cai Z., Pan T. Detection and quantification of glycosylated queuosine modified tRNAs by acid denaturing and APB gels. RNA. 2020; 26:1291–1298. 32439717 10.1261/rna.075556.120 PMC7430669

[13] Siard T.J. , Katze J.R., Farkas W.R. Queuine is incorporated into brain transfer RNA. Neurochem. Res. 1989; 14:1159–1164. 2594145 10.1007/BF00965624

[14] Richard P. , Kozlowski L., Guillorit H., Garnier P., McKnight N.C., Danchin A., Maniere X. Queuine, a bacterial-derived hypermodified nucleobase, shows protection in in vitro models of neurodegeneration. PLoS One. 2021; 16:e0253216. 34379627 10.1371/journal.pone.0253216 PMC8357117

[15] Igloi G.L. , Kossel H. Affinity electrophoresis for monitoring terminal phosphorylation and the presence of queuosine in RNA. Application of polyacrylamide containing a covalently bound boronic acid. Nucleic Acids Res. 1985; 13:6881–6898. 2414733 10.1093/nar/13.19.6881 PMC322011

[16] Costa A. , Pais de Barros J.P., Keith G., Baranowski W., Desgres J. Determination of queuosine derivatives by reverse-phase liquid chromatography for the hypomodification study of Q-bearing tRNAs from various mammal liver cells. J. Chromatogr. B. Analyt. Technol. Biomed. Life Sci. 2004; 801:237–247. 10.1016/j.jchromb.2003.11.022 14751792

[17] Zaborske J.M. , DuMont V.L., Wallace E.W., Pan T., Aquadro C.F., Drummond D.A. A nutrient-driven tRNA modification alters translational fidelity and genome-wide protein coding across an animal genus. PLoS Biol. 2014; 12:e1002015. 25489848 10.1371/journal.pbio.1002015 PMC4260829

[18] Boccaletto P. , Stefaniak F., Ray A., Cappannini A., Mukherjee S., Purta E., Kurkowska M., Shirvanizadeh N., Destefanis E., Groza P. et al. MODOMICS: a database of RNA modification pathways. 2021 update. Nucleic Acids Res. 2022; 50:D231–D235. 34893873 10.1093/nar/gkab1083 PMC8728126

[19] Johansson M.J.O. , Xu F., Bystrom A.S. Elongator-a tRNA modifying complex that promotes efficient translational decoding. Biochim. Biophys. Acta. Gene. Regul. Mech. 2018; 1861:401–408. 29170010 10.1016/j.bbagrm.2017.11.006

[20] Vangaveti S. , Cantara W.A., Spears J.L., DeMirci H., Murphy F.V.t., Ranganathan S.V., Sarachan K.L., Agris P.F. A structural basis for restricted codon recognition mediated by 2-thiocytidine in tRNA containing a Wobble position inosine. J. Mol. Biol. 2020; 432:913–929. 31945376 10.1016/j.jmb.2019.12.016 PMC7102896

[21] Kramer G.F. , Baker J.C., Ames B.N. Near-UV stress in *Salmonella typhimurium*: 4-thiouridine in tRNA, ppGpp, and ApppGpp as components of an adaptive response. J. Bacteriol. 1988; 170:2344–2351. 3283108 10.1128/jb.170.5.2344-2351.1988 PMC211128

[22] Ryvkin P. , Leung Y.Y., Silverman I.M., Childress M., Valladares O., Dragomir I., Gregory B.D., Wang L.S. HAMR: high-throughput annotation of modified ribonucleotides. RNA. 2013; 19:1684–1692. 24149843 10.1261/rna.036806.112 PMC3884653

[23] Motorin Y. , Muller S., Behm-Ansmant I., Branlant C. Identification of modified residues in RNAs by reverse transcription-based methods. Methods Enzymol. 2007; 425:21–53. 17673078 10.1016/S0076-6879(07)25002-5

[24] Clark W.C. , Evans M.E., Dominissini D., Zheng G., Pan T. tRNA base methylation identification and quantification via high-throughput sequencing. RNA. 2016; 22:1771–1784. 27613580 10.1261/rna.056531.116 PMC5066629

[25] Watkins C.P. , Zhang W., Wylder A.C., Katanski C.D., Pan T. A multiplex platform for small RNA sequencing elucidates multifaceted tRNA stress response and translational regulation. Nat. Commun. 2022; 13:2491. 35513407 10.1038/s41467-022-30261-3 PMC9072684

[26] Langmead B. , Salzberg S.L. Fast gapped-read alignment with Bowtie 2. Nat. Methods. 2012; 9:357–359. 22388286 10.1038/nmeth.1923 PMC3322381

[27] Danecek P. , Bonfield J.K., Liddle J., Marshall J., Ohan V., Pollard M.O., Whitwham A., Keane T., McCarthy S.A., Davies R.M. et al. Twelve years of SAMtools and BCFtools. Gigascience. 2021; 10:giab008. 33590861 10.1093/gigascience/giab008 PMC7931819

[28] Lewis J.A. , Ames B.N. Histidine regulation in Salmonella typhimurium. XI. The percentage of transfer RNA His charged in vivo and its relation to the repression of the histidine operon. J. Mol. Biol. 1972; 66:131–142. 4339187 10.1016/s0022-2836(72)80011-1

[29] Dittmar K.A. , Sorensen M.A., Elf J., Ehrenberg M., Pan T. Selective charging of tRNA isoacceptors induced by amino-acid starvation. EMBO Rep. 2005; 6:151–157. 15678157 10.1038/sj.embor.7400341 PMC1299251

[30] Evans M.E. , Clark W.C., Zheng G., Pan T. Determination of tRNA aminoacylation levels by high-throughput sequencing. Nucleic Acids Res. 2017; 45:e133. 28586482 10.1093/nar/gkx514 PMC5737633

[31] Behrens A. , Rodschinka G., Nedialkova D.D. High-resolution quantitative profiling of tRNA abundance and modification status in eukaryotes by mim-tRNAseq. Mol. Cell. 2021; 81:1802–1815. 33581077 10.1016/j.molcel.2021.01.028 PMC8062790

[32] Kasai H. , Kuchino Y., Nihei K., Nishimura S. Distribution of the modified nucleoside Q and its derivatives in animal and plant transfer RNA’s. Nucleic Acids Res. 1975; 2:1931–1939. 1187350 10.1093/nar/2.10.1931 PMC343557

[33] Kasai H. , Oashi Z., Harada F., Nishimura S., Oppenheimer N.J., Crain P.F., Liehr J.G., von Minden D.L., McCloskey J.A. Structure of the modified nucleoside Q isolated from *Escherichia coli* transfer ribonucleic acid. 7-(4,5-cis-dihydroxy-1-cyclopenten-3-ylaminomethyl)-7-deazaguanosine. Biochemistry. 1975; 14:4198–4208. 1101947 10.1021/bi00690a008

[34] Suzuki T. , Yashiro Y., Kikuchi I., Ishigami Y., Saito H., Matsuzawa I., Okada S., Mito M., Iwasaki S., Ma D. et al. Complete chemical structures of human mitochondrial tRNAs. Nat. Commun. 2020; 11:4269. 32859890 10.1038/s41467-020-18068-6 PMC7455718

[35] Grosjean H.J. , de Henau S., Crothers D.M. On the physical basis for ambiguity in genetic coding interactions. Proc. Natl. Acad. Sci. U.S.A. 1978; 75:610–614. 273223 10.1073/pnas.75.2.610 PMC411305

[36] Morris R.C. , Brown K.G., Elliott M.S. The effect of queuosine on tRNA structure and function. J. Biomol. Struct. Dyn. 1999; 16:757–774. 10217448 10.1080/07391102.1999.10508291

[37] Ehrenhofer-Murray A.E. Cross-talk between Dnmt2-dependent tRNA methylation and queuosine modification. Biomolecules. 2017; 7:E14. 28208632 10.3390/biom7010014 PMC5372726

[38] Wu X. , Li Z., Chen X.X., Fossey J.S., James T.D., Jiang Y.B. Selective sensing of saccharides using simple boronic acids and their aggregates. Chem. Soc. Rev. 2013; 42:8032–8048. 23860576 10.1039/c3cs60148j

[39] Li X. , Xiong X., Wang K., Wang L., Shu X., Ma S., Yi C. Transcriptome-wide mapping reveals reversible and dynamic N(1)-methyladenosine methylome. Nat. Chem. Biol. 2016; 12:311–316. 26863410 10.1038/nchembio.2040

[40] Zhou H. , Rauch S., Dai Q., Cui X., Zhang Z., Nachtergaele S., Sepich C., He C., Dickinson B.C. Evolution of a reverse transcriptase to map N(1)-methyladenosine in human messenger RNA. Nat. Methods. 2019; 16:1281–1288. 31548705 10.1038/s41592-019-0550-4 PMC6884687

[41] Sochacka E. , Kraszewska K., Sochacki M., Sobczak M., Janicka M., Nawrot B. The 2-thiouridine unit in the RNA strand is desulfured predominantly to 4-pyrimidinone nucleoside under in vitro oxidative stress conditions. Chem. Commun. (Camb.). 2011; 47:4914–4916. 21431224 10.1039/c1cc10973a

[42] Sudalai A. , Khenkin A., Neumann R. Sodium periodate mediated oxidative transformations in organic synthesis. Org. Biomol. Chem. 2015; 13:4374–4394. 25768201 10.1039/c5ob00238a

[43] Wang J. , Shang J., Qin Z., Tong A., Xiang Y. Selective and sensitive fluorescence ‘turn-on’ detection of 4-thiouridine in nucleic acids via oxidative amination. Chem. Commun. (Camb.). 2019; 55:13096–13099. 31612162 10.1039/c9cc06312a

[44] Schwartz M.H. , Wang H., Pan J.N., Clark W.C., Cui S., Eckwahl M.J., Pan D.W., Parisien M., Owens S.M., Cheng B.L. et al. Microbiome characterization by high-throughput transfer RNA sequencing and modification analysis. Nat. Commun. 2018; 9:5353. 30559359 10.1038/s41467-018-07675-z PMC6297222

[45] Bouvier D. , Labessan N., Clemancey M., Latour J.M., Ravanat J.L., Fontecave M., Atta M. TtcA a new tRNA-thioltransferase with an Fe-S cluster. Nucleic Acids Res. 2014; 42:7960–7970. 24914049 10.1093/nar/gku508 PMC4081106

[46] Helm M. , Motorin Y. Detecting RNA modifications in the epitranscriptome: predict and validate. Nat. Rev. Genet. 2017; 18:275–291. 28216634 10.1038/nrg.2016.169

[47] Werner S. , Schmidt L., Marchand V., Kemmer T., Falschlunger C., Sednev M.V., Bec G., Ennifar E., Hobartner C., Micura R. et al. Machine learning of reverse transcription signatures of variegated polymerases allows mapping and discrimination of methylated purines in limited transcriptomes. Nucleic Acids Res. 2020; 48:3734–3746. 32095818 10.1093/nar/gkaa113 PMC7144921

