# Supplementary material for: Analysis of queuosine and 2-thio tRNA modifications by high throughput sequencing
Source: Nucleic Acids Res. 2022 Jun 17;50(17):e99. doi: 10.1093/nar/gkac517 (PMC9508811; doi:10.1093/nar/gkac517)
Supplement: gkac517_Supplemental_File [file gkac517_supplemental_file.pptx]

## Slide 1
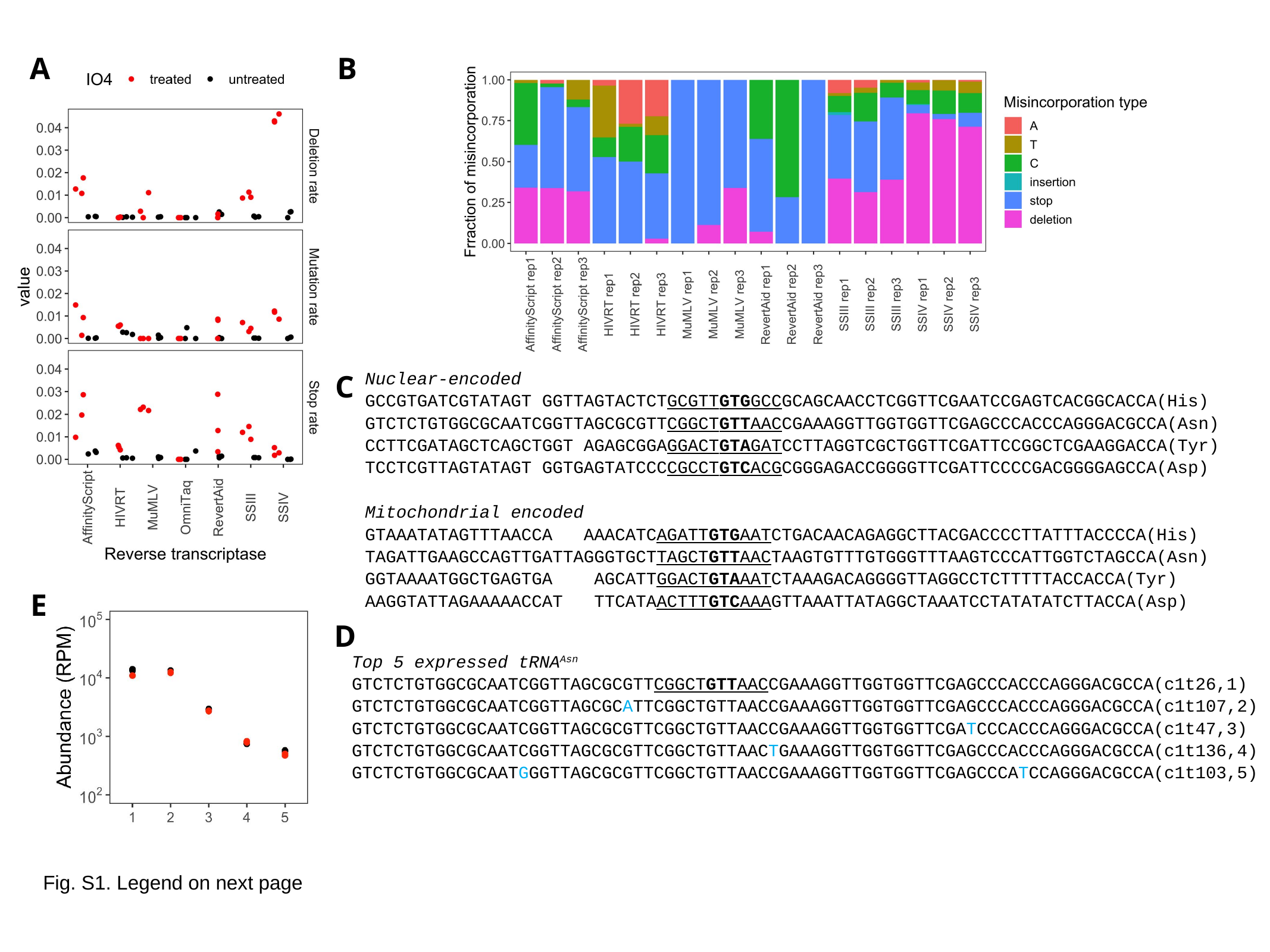

A
B
Nuclear-encoded
GCCGTGATCGTATAGT GGTTAGTACTCTGCGTTGTGGCCGCAGCAACCTCGGTTCGAATCCGAGTCACGGCACCA(His)
GTCTCTGTGGCGCAATCGGTTAGCGCGTTCGGCTGTTAACCGAAAGGTTGGTGGTTCGAGCCCACCCAGGGACGCCA(Asn)
CCTTCGATAGCTCAGCTGGT AGAGCGGAGGACTGTAGATCCTTAGGTCGCTGGTTCGATTCCGGCTCGAAGGACCA(Tyr)
TCCTCGTTAGTATAGT GGTGAGTATCCCCGCCTGTCACGCGGGAGACCGGGGTTCGATTCCCCGACGGGGAGCCA(Asp)
Mitochondrial encoded
GTAAATATAGTTTAACCA   AAACATCAGATTGTGAATCTGACAACAGAGGCTTACGACCCCTTATTTACCCCA(His)
TAGATTGAAGCCAGTTGATTAGGGTGCTTAGCTGTTAACTAAGTGTTTGTGGGTTTAAGTCCCATTGGTCTAGCCA(Asn)
GGTAAAATGGCTGAGTGA    AGCATTGGACTGTAAATCTAAAGACAGGGGTTAGGCCTCTTTTTACCACCA(Tyr)
AAGGTATTAGAAAAACCAT   TTCATAACTTTGTCAAAGTTAAATTATAGGCTAAATCCTATATATCTTACCA(Asp)
C
E
D
Top 5 expressed tRNAAsn
GTCTCTGTGGCGCAATCGGTTAGCGCGTTCGGCTGTTAACCGAAAGGTTGGTGGTTCGAGCCCACCCAGGGACGCCA(c1t26,1)
GTCTCTGTGGCGCAATCGGTTAGCGCATTCGGCTGTTAACCGAAAGGTTGGTGGTTCGAGCCCACCCAGGGACGCCA(c1t107,2)
GTCTCTGTGGCGCAATCGGTTAGCGCGTTCGGCTGTTAACCGAAAGGTTGGTGGTTCGATCCCACCCAGGGACGCCA(c1t47,3)
GTCTCTGTGGCGCAATCGGTTAGCGCGTTCGGCTGTTAACTGAAAGGTTGGTGGTTCGAGCCCACCCAGGGACGCCA(c1t136,4)
GTCTCTGTGGCGCAATGGGTTAGCGCGTTCGGCTGTTAACCGAAAGGTTGGTGGTTCGAGCCCATCCAGGGACGCCA(c1t103,5)
Fig. S1. Legend on next page

## Slide 2
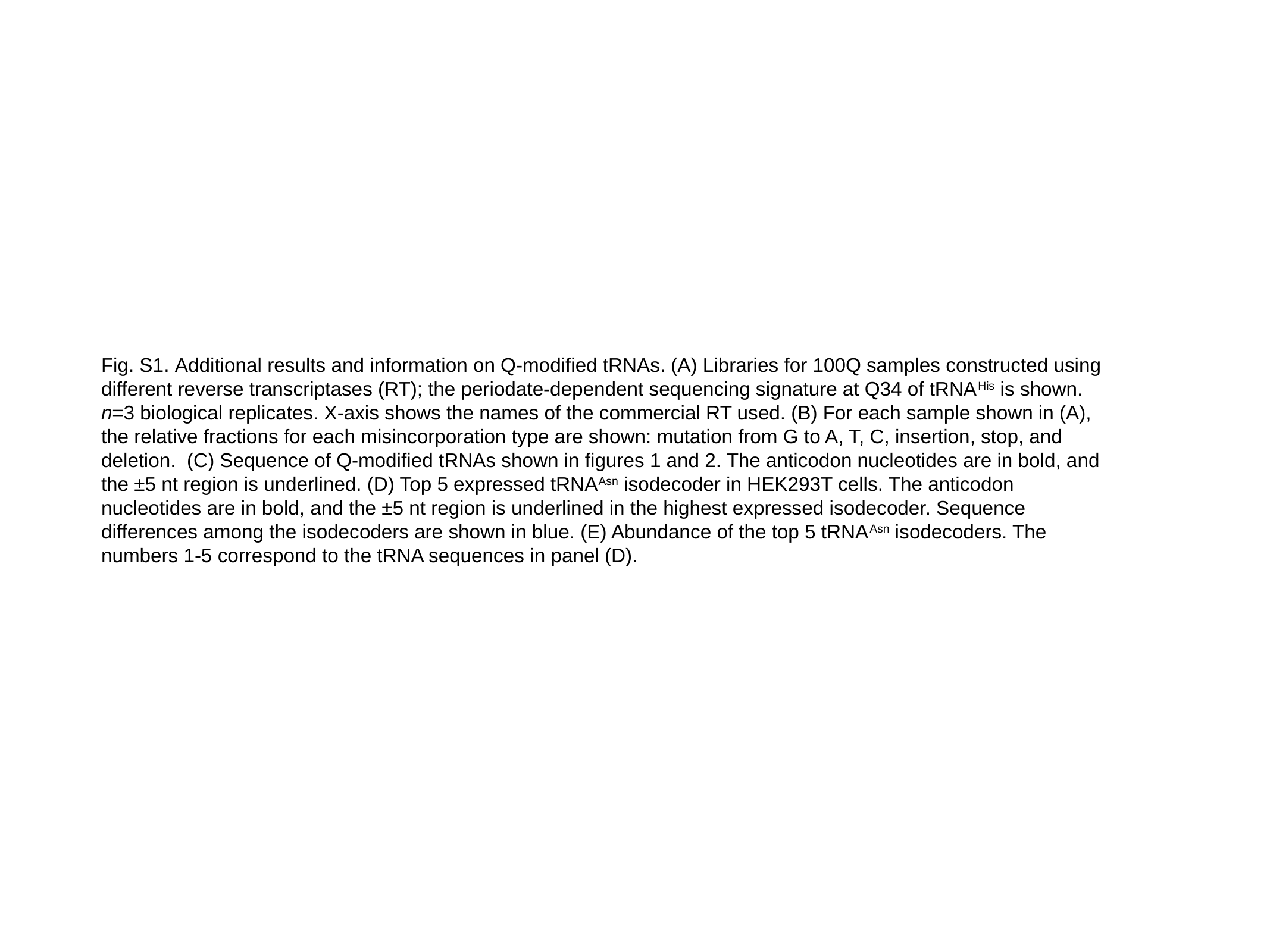

Fig. S1. Additional results and information on Q-modified tRNAs. (A) Libraries for 100Q samples constructed using different reverse transcriptases (RT); the periodate-dependent sequencing signature at Q34 of tRNAHis is shown. n=3 biological replicates. X-axis shows the names of the commercial RT used. (B) For each sample shown in (A), the relative fractions for each misincorporation type are shown: mutation from G to A, T, C, insertion, stop, and deletion.  (C) Sequence of Q-modified tRNAs shown in figures 1 and 2. The anticodon nucleotides are in bold, and the ±5 nt region is underlined. (D) Top 5 expressed tRNAAsn isodecoder in HEK293T cells. The anticodon nucleotides are in bold, and the ±5 nt region is underlined in the highest expressed isodecoder. Sequence differences among the isodecoders are shown in blue. (E) Abundance of the top 5 tRNAAsn isodecoders. The numbers 1-5 correspond to the tRNA sequences in panel (D).

## Slide 3
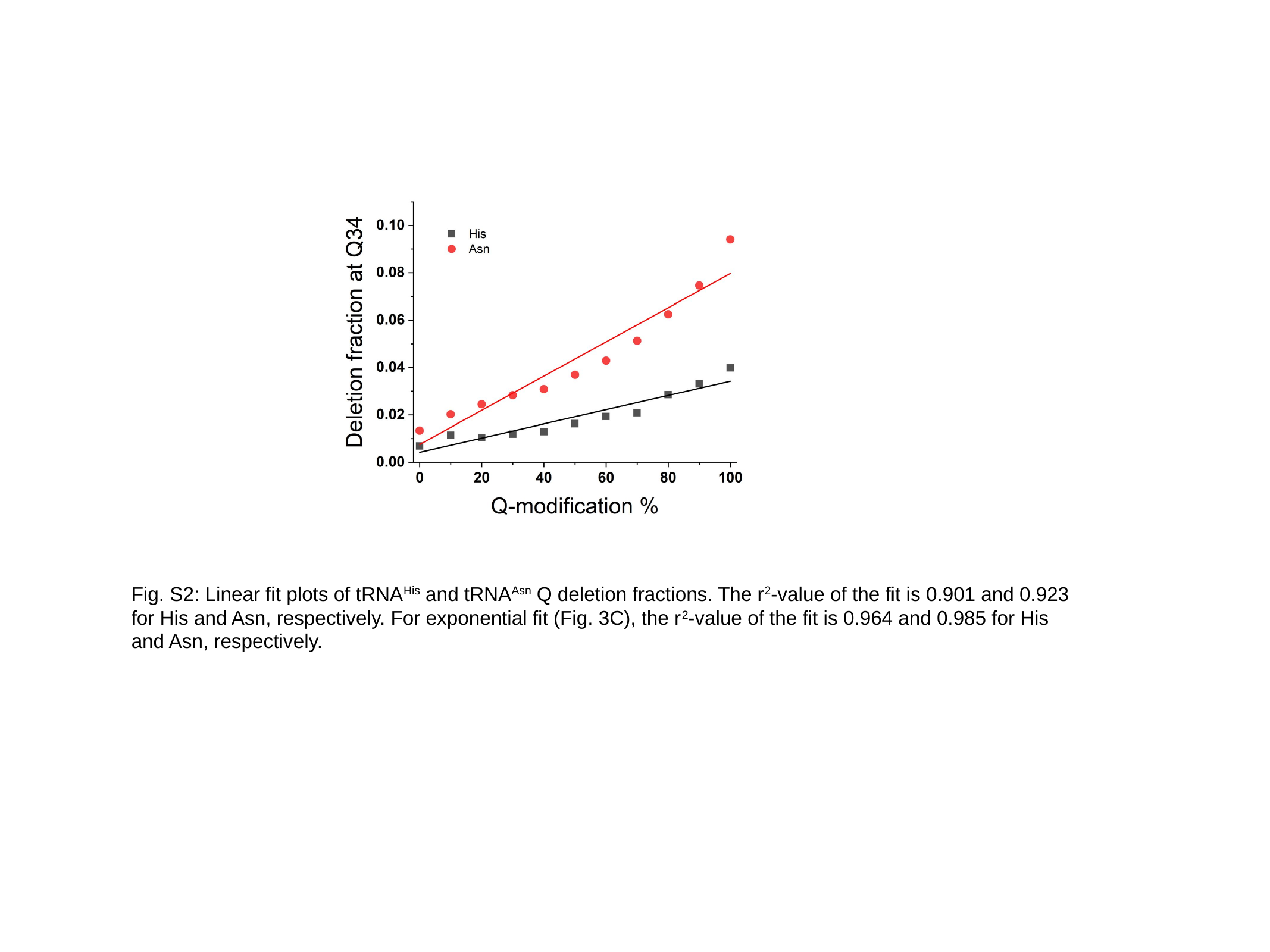

Fig. S2: Linear fit plots of tRNAHis and tRNAAsn Q deletion fractions. The r2-value of the fit is 0.901 and 0.923 for His and Asn, respectively. For exponential fit (Fig. 3C), the r2-value of the fit is 0.964 and 0.985 for His and Asn, respectively.

## Slide 4
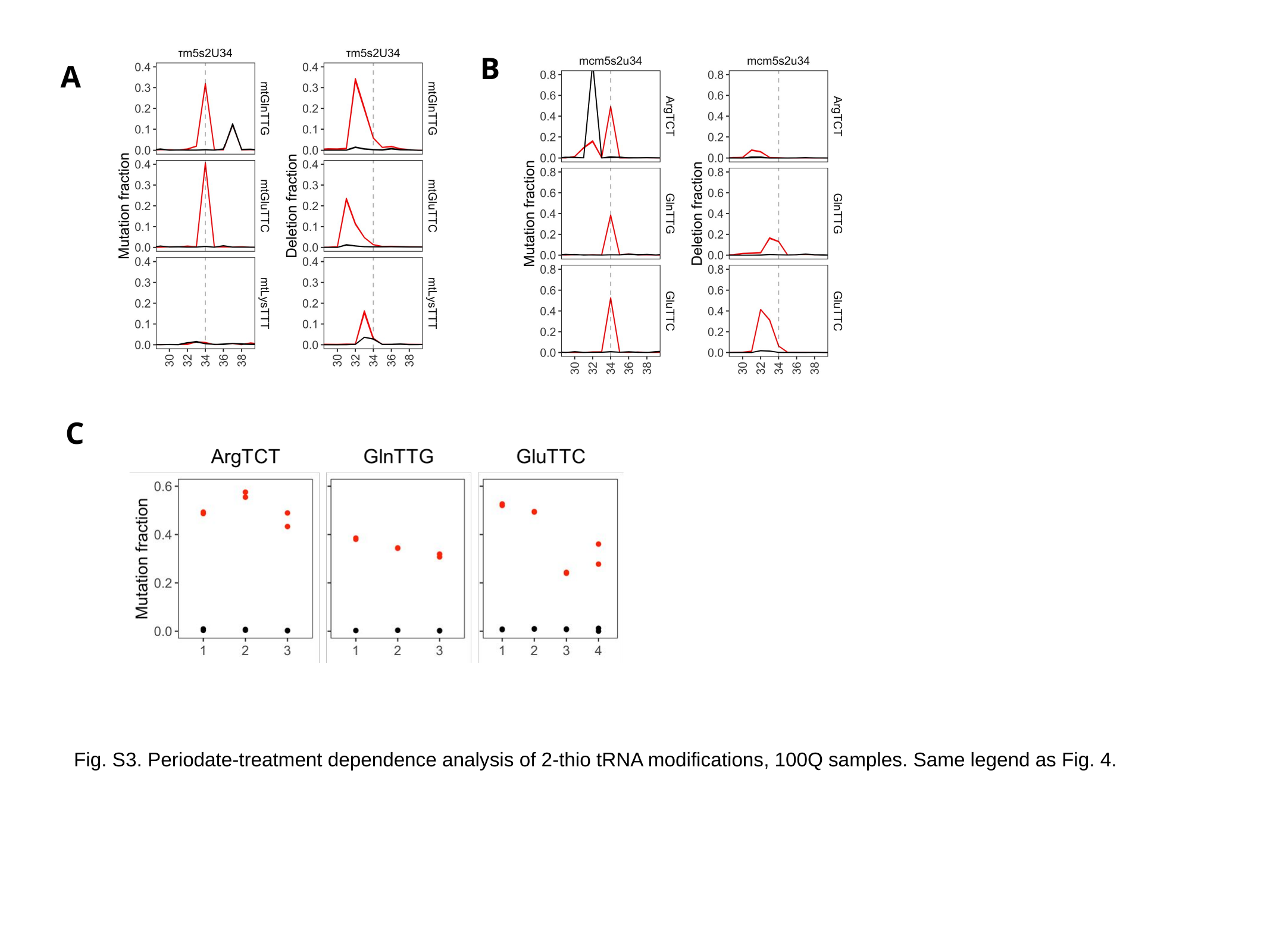

B
A
C
Fig. S3. Periodate-treatment dependence analysis of 2-thio tRNA modifications, 100Q samples. Same legend as Fig. 4.

## Slide 5
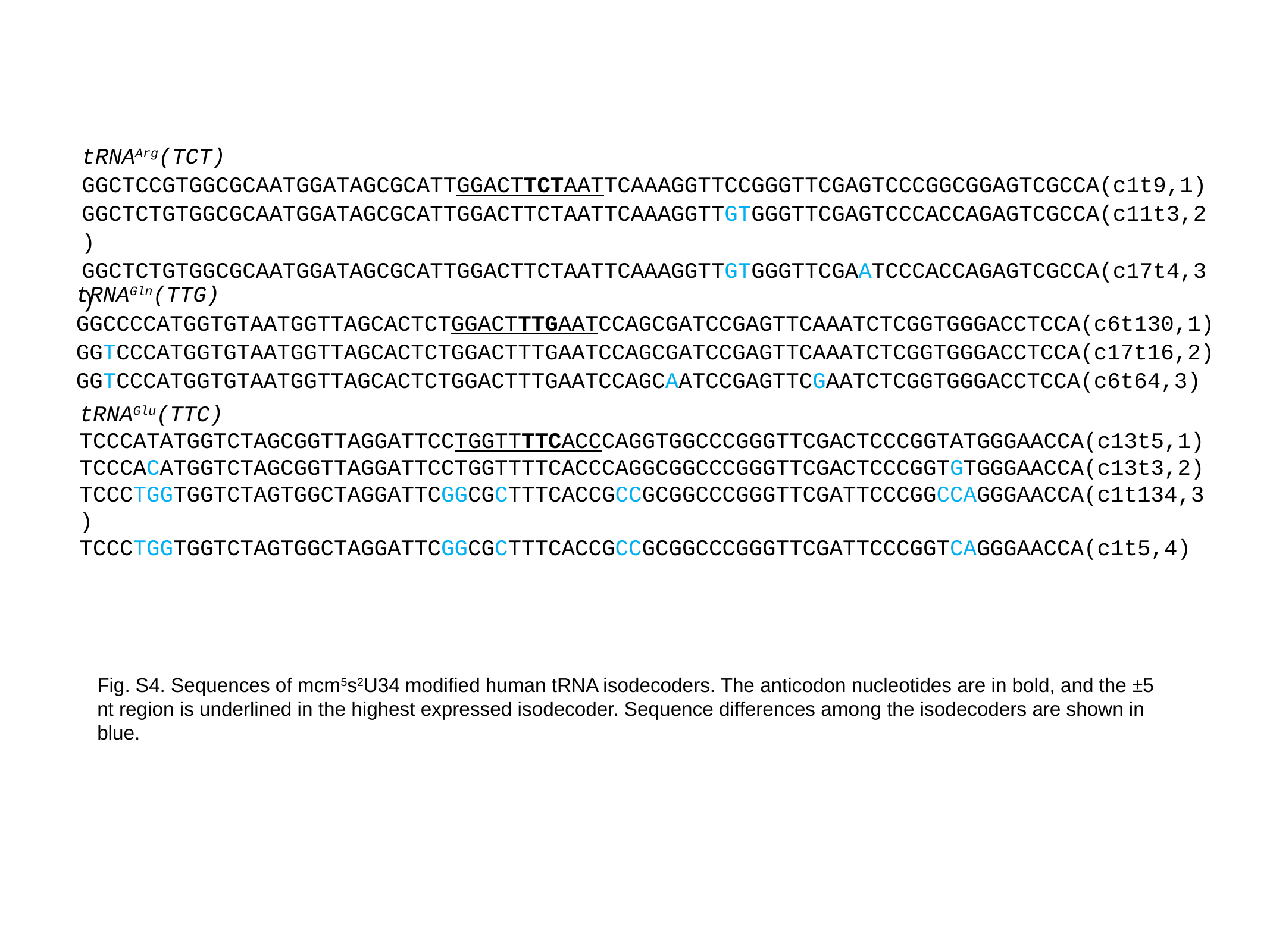

tRNAArg(TCT) GGCTCCGTGGCGCAATGGATAGCGCATTGGACTTCTAATTCAAAGGTTCCGGGTTCGAGTCCCGGCGGAGTCGCCA(c1t9,1)
GGCTCTGTGGCGCAATGGATAGCGCATTGGACTTCTAATTCAAAGGTTGTGGGTTCGAGTCCCACCAGAGTCGCCA(c11t3,2)
GGCTCTGTGGCGCAATGGATAGCGCATTGGACTTCTAATTCAAAGGTTGTGGGTTCGAATCCCACCAGAGTCGCCA(c17t4,3)
tRNAGln(TTG) GGCCCCATGGTGTAATGGTTAGCACTCTGGACTTTGAATCCAGCGATCCGAGTTCAAATCTCGGTGGGACCTCCA(c6t130,1)
GGTCCCATGGTGTAATGGTTAGCACTCTGGACTTTGAATCCAGCGATCCGAGTTCAAATCTCGGTGGGACCTCCA(c17t16,2)
GGTCCCATGGTGTAATGGTTAGCACTCTGGACTTTGAATCCAGCAATCCGAGTTCGAATCTCGGTGGGACCTCCA(c6t64,3)
tRNAGlu(TTC) TCCCATATGGTCTAGCGGTTAGGATTCCTGGTTTTCACCCAGGTGGCCCGGGTTCGACTCCCGGTATGGGAACCA(c13t5,1)
TCCCACATGGTCTAGCGGTTAGGATTCCTGGTTTTCACCCAGGCGGCCCGGGTTCGACTCCCGGTGTGGGAACCA(c13t3,2)
TCCCTGGTGGTCTAGTGGCTAGGATTCGGCGCTTTCACCGCCGCGGCCCGGGTTCGATTCCCGGCCAGGGAACCA(c1t134,3)
TCCCTGGTGGTCTAGTGGCTAGGATTCGGCGCTTTCACCGCCGCGGCCCGGGTTCGATTCCCGGTCAGGGAACCA(c1t5,4)
Fig. S4. Sequences of mcm5s2U34 modified human tRNA isodecoders. The anticodon nucleotides are in bold, and the ±5 nt region is underlined in the highest expressed isodecoder. Sequence differences among the isodecoders are shown in blue.

## Slide 6
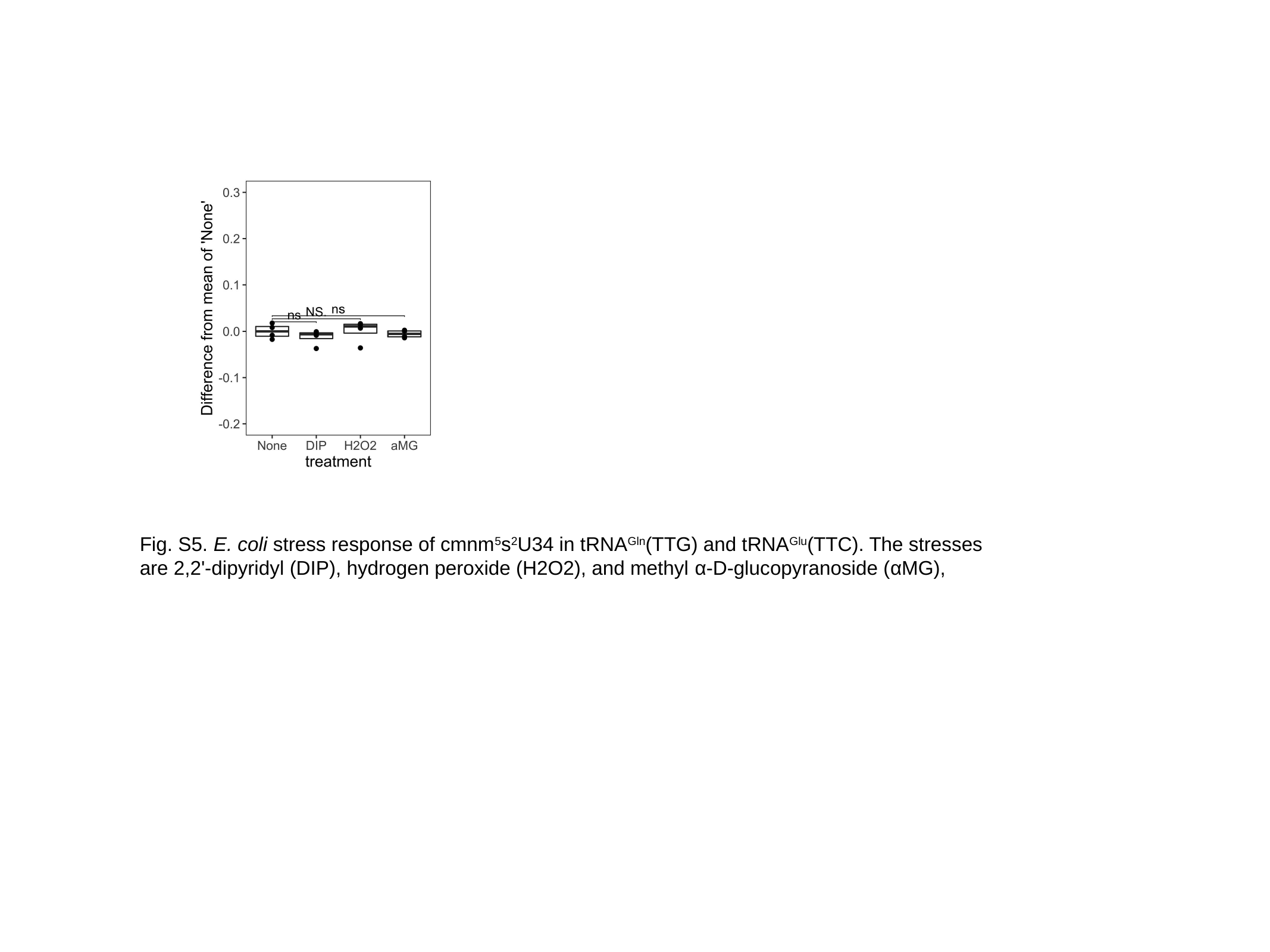

Fig. S5. E. coli stress response of cmnm5s2U34 in tRNAGln(TTG) and tRNAGlu(TTC). The stresses are 2,2'-dipyridyl (DIP), hydrogen peroxide (H2O2), and methyl α-D-glucopyranoside (αMG),
